# Routine Health Facility and Community Information Systems: Creating an Information Use Culture

**DOI:** 10.9745/GHSP-D-17-00319

**Published:** 2017-09-27

**Authors:** Theo Lippeveld

**Affiliations:** a John Snow, Inc., Data Use Partnership Project, Addis Ababa, Ethiopia.

## Abstract

Substantial progress has been made to strengthen health information systems, with most efforts focusing on digitization, improving data quality and analysis, and *identifying problems*. But the ultimate goal is *using* information to *solve problems*, which requires building an information use culture over time. How? Human-centered design, role modeling by senior managers in use of data, and incentive-based systems hold considerable promise.

See related articles by Biemba et al., Hazel et al., and O'Hagan et al.

Since the 1990s, knowledge and understanding of the role of health information on global health systems have markedly improved. Despite this, use of information for evidence-based decision making is still very weak in most low- and middle-income countries (LMICs), and particularly for data produced by health facility and community information systems, also called routine health information systems (RHISs). Ill-defined information needs, major data quality issues, and centralization and fragmentation of health information systems are some of the root causes, leading to poor quality and use of routine information at all levels.

The Paris Declaration and initiatives such as the Health Metrics Network, the Millennium Development Goals, and the Sustainable Development Goals have triggered governments of LMICs to make the development of well-performing RHISs a high priority. In June 2015, at the Measurement and Accountability for Health Summit, the U.S. Agency for International Development, the World Health Organization (WHO), and the World Bank called for action “to improve health facility and community information systems including disease and risk surveillance and financial and health workforce accounts, empowering decision makers at all levels with real-time access to information.”^1^

Based on this Health Summit, the Health Data Collaborative was created, which has a special focus on improving RHIS performance. WHO and the MEASURE Evaluation project, in collaboration with many university partners, have developed an RHIS standard curriculum for health managers and care providers.^2^ Many LMICs in Africa and Asia have made strengthening of RHISs one of their main health systems strengthening priorities.

In this issue of GHSP, 3 articles have been published about RHIS performance improvement in Malawi and Zambia:

- Richael O'Hagan et al.^3^ “National assessment of data quality and associated systems-level factors in Malawi”
- Elizabeth Hazel et al.^4^ “Using data to improve programs: assessment of a data quality and use intervention package for integrated community case management in Malawi”
- Godfrey Biemba et al.^5^ “A mobile-based community health management information system for community health workers and their supervisors in 2 districts of Zambia”

## DATA QUALITY ASSESSMENTS VS. DATA QUALITY ASSURANCE SYSTEMS

The O'Hagan article^3^ focuses on data quality assessment of the facility-based health management information system (HMIS) in Malawi, using a customized set of data quality assessment (DQA) tools developed by WHO. The findings indicate weaknesses in data quality based on lack of data quality assurance systems and unreliable supervision. The authors advise LMICs to regularly undertake such types of assessments. The article puts a lot of emphasis on systemic issues of data quality, which is a very welcome viewpoint. Previous data quality assessment tools were very program-specific, but the Data Quality Review tool, recently developed by WHO and MEASURE Evaluation, has a health systems component. Also, a district version of the Data Quality Review tool is under development. Most of all, rather than assessing data quality, priority should be given to setting up institutionalized mechanisms of data quality assurance at all levels of the health system, thereby addressing in a preventive way the production of low-quality data.

Priority should be given to setting up institutionalized mechanisms of data quality assurance at all levels of the health system.

## COMMUNITY-BASED HEALTH INFORMATION SYSTEMS

The 2 other articles in this issue focus on community-based health information systems (CHIS or C-HMIS). In recent years, many LMICs have developed community-based health services, which are delivered by community health workers in close connection to primary care facilities. These community health systems address the existing dearth of skilled workforce by training community health workers and volunteers to deliver simple health care services as well as behavioral change interventions, and expanding as such the coverage of the health services. Key to such efforts is the development and strengthening of CHISs as an integral part of facility- and community-based health information systems to improve the availability, accessibility, quality, and use of community health data.

The Hazel article^4^ discusses the development of a data quality and use package for integrated community case management (iCCM) data in Malawi. This training package is based on MEASURE Evaluation guidelines for data analysis and interpretation. While the data quality and use package allows for better data analysis using data visualization templates, it addresses only iCCM data, which is a small part of the routine data for community-based health services. Hopefully, this approach can be expanded to other community services.

The Biemba article^5^ examines the development of a community version of the open-source District Health Information System 2 (DHIS 2) application in Zambia. Using mobile technology (simple-feature phones), community-based data can be reported into the facility-based DHIS 2 database, allowing for better integration of facility- and community-based data. However, there is a need for ongoing technical support to address the hardware and software challenges faced by the community health workers.

## THE ULTIMATE GOAL: TRANSLATING DATA INTO ACTION

As illustrated in the 3 articles, substantial progress has been made in improving RHIS performance and developing relevant CHISs, but many challenges remain such as fragmentation and disjointed efforts to strengthen community- and facility-based health information systems. Greater collaboration, coordination, and joint action are needed at global and particularly country levels to address these challenges, accelerate progress, and achieve national health priorities.

Substantial progress has been made in improving routine health information system performance and developing relevant community health information systems, but many challenges remain.

Yet most efforts to strengthen health facility and community health information systems are focused on digitization, improving data quality and data analysis, and identifying problems. But the ultimate goal of RHISs is that information is used *to solve problems* and to improve access to and delivery of quality health services. This last step of translating data into action is the most challenging, and many barriers have been identified leading to poor use of data for action, such as poor data quality, poor access to data, lack of capacity of health managers and providers in core competencies for data use, and poor identification of information needs.^6^

The ultimate goal of routine health information systems is that information is used to solve problems.

### Behavioral Barriers to Creating an Information Use Culture

While most of these barriers to data use are technical issues that can be addressed by technical solutions, many barriers are linked to organizational and behavioral factors as explained in the PRISM framework.^7^ The decision-making and problem-solving behavior of data users can heavily influence the ultimate use of data for service delivery improvements. Both data producers and users function in an organizational context that can support or hinder them to use information for action. An example of negative organizational behavior is the pressure exerted by senior health managers on district health managers and care providers to reach unrealistic service delivery targets, leading to false reporting and denial of existing service delivery problems. An excellent example of positive organizational behavior has been published in the *Quarterly Journal of Economics* by Bjorkman et al.^8^ The authors, through a randomized field experiment in 9 districts in Uganda, document how community monitoring of health service delivery data, as well as active participation and accountability by the communities, led to large increases in utilization of services and improved health outcomes.

Many barriers to data use are linked to organizational and behavioral factors.

RHIS strengthening therefore involves *building an information culture* where information is valued at all levels of the health system. The challenge of creating a culture of data use is that this is a behavioral change intervention, both at the individual and organizational level. As with all behavior change interventions, the time span to create new perceptions, attitudes, and skills of users related to the value of information ranges from 10 years to a “generation” (25 years), so way beyond the classical 5-year period of most projects. Yet cultural change, once it has been established, will ultimately lead to sustained data use at all levels of the health system.

Strengthening routine health information systems involves building an information culture where information is valued at all health systems levels.

### Potential Solutions to Creating an Information Use Culture

The question is how to build an information use culture. Recently, human-centered design (HCD) has been used increasingly in the private sector for product and technology development as an approach to better understand the user needs and involve them early on in the design of solutions. HCD is a collaborative problem-solving approach that provides broadly applicable methods of developing an in-depth understanding of human behavior.^9^ It involves the process of understanding the “how” and the “why” of a problem. This approach can be adopted not only to create products and technologies but also to develop systems, programs, and services that are most needed by the users and that are most appropriate in the given context to maximize impact and outcomes. Therefore, the HCD approach and methods, as an organizational behavioral intervention, could be applied in establishing a culture of information, together with other promising interventions such as role modeling by senior managers to promote use of data at the district level and below (as in Ethiopia), as well as incentive-based systems to promote use of information including performance-based financing schemes (e.g., Benin, Liberia, Rwanda); allocation of resources based on HMIS indicator results (e.g., Brazil); and use of information as criteria for annual performance appraisals.

Human-centered design could be used to help build an information culture.

Many of these innovative approaches to promote information use at all levels will be tested in the coming years in “Data Use Partnerships” (DUP), which have been established by the governments of Ethiopia, Malawi, and Tanzania with funding by the Bill & Melinda Gates Foundation, and with technical support by various implementing partners. One of the main principles of DUP is the promotion of country ownership and accountability for the national HMIS, and less dependency on donor-driven projects, so as to ensure a long-term investment in building high-performing HMISs and in the establishment of a sustainable information culture.

It is hoped that many countries in Africa and Asia will set up comparable initiatives and establish an information culture with institutionalized mechanisms for use of RHIS information for improved service delivery at all levels of the health system.
